# Retraction Note: BMP2 expression in oral squamous cell carcinoma and its effects on SCC9 cell biological behavior

**DOI:** 10.1038/s41598-026-42309-1

**Published:** 2026-03-09

**Authors:** Fuao Xing, Yimin Liu, Faming Tian, Xiaoli Hou, Qiangqiang Lian, Yunpeng Hu, Lei Xing, JingYuan Gao, Xinhao Fan

**Affiliations:** 1 North China University of Science and Technology, Tangshan, 063000 HeBei China; 2 Affiliated Hospital of North China University of Science and Technology, Tangshan, 063000 HeBei China; 3 Affiliated Kailuan General Hospital of North China University of Science and Technology, 57 Xinhua East Road, Tangshan, 063000 HeBei China; 4 Second Hospital of Tangshan, Tangshan, 063000 HeBei China; 5 School of Dental Sciences, Universiti Sains Malaysia, Kubang Kerian, 16150 Kelantan Malaysia

Retraction of: *Scientific Reports* 10.1038/s41598-025-96274-2, published online 04 April 2025

The Editors have retracted this Article.

After publication, concerns were raised that images had been duplicated between panels in Figure 4. The Editors requested all original data underlying the study and found that the metadata for Figure 4A indicated the time course of the siRNA wound-healing assays was inaccurately reported. The Editors no longer have confidence in the validity of this Article.

All Authors agree with the retraction and its wording.

